# Thioredoxin-Interacting Protein Gene Expression via MondoA Is Rapidly and Transiently Suppressed during Inflammatory Responses

**DOI:** 10.1371/journal.pone.0059026

**Published:** 2013-03-08

**Authors:** Yasuyoshi Kanari, Yuki Sato, Satoru Aoyama, Tatsushi Muta

**Affiliations:** 1 Laboratory of Cell Recognition and Response, Graduate School of Life Sciences, Tohoku University, Sendai, Miyagi, Japan; 2 Global Center of Excellence Program, Center for Ecosystem Management Adapting to Global Change, Sendai, Miyagi, Japan; 3 Department of Biology, Faculty of Science, Tohoku University, Sendai, Miyagi, Japan; University of Nebraska – Lincoln, United States of America

## Abstract

Whereas accumulating evidence indicates that a number of inflammatory genes are induced by activation of nuclear factor-κB and other transcription factors, less is known about genes that are suppressed by proinflammatory stimuli. Here we show that expression of thioredoxin-interacting protein (*Txnip*) is dramatically suppressed both in mRNA and protein levels upon stimulation with lipopolysaccharide in mouse and human macrophages. In addition to lipopolysaccharide, a Toll-like receptor 4 ligand, stimulation with other Toll-like receptor ligands such as CpG DNA also suppressed *Txnip* expression. Not only the Toll-like receptor ligands, but also other proinflammatory stimulators, such as interleukin-1β and tumor necrosis factor-α elicited the similar response in fibroblasts. Suppression of *Txnip* by lipopolysaccharide is accompanied by a decrease of the glucose sensing transcription factor MondoA in the nuclei and dissociation of the MondoA:Mlx complex that bound to the carbohydrate-response elements in the *Txnip* promoter in unstimulated cells. Lipopolysaccharide-mediated decrease of nuclear MondoA was inhibited in the presence of 2-deoxyglucose. Furthermore, blockage of glyceraldehyde-3-phosphate dehydrogenase by iodoacetate alleviated the suppression of *Txnip* mRNA by lipopolysaccharide, suggesting the involvement of glucose-metabolites in the regulation. Since *Txnip* is implicated in the regulation of glucose metabolism, this observation links between inflammatory responses and metabolic regulation.

## Introduction

A variety of microbial substances so-called pathogen-associated molecular patterns are recognized by Toll-like receptors (TLRs) and other pattern recognition receptors; binding stimulates dynamic alteration of expression profiles for production of inflammatory cytokines and chemokines [1]. During this process, expression of a very large number of genes is induced by activation of nuclear factor-κB, activator protein-1 and other transcription factors. In contrast to the induction of inflammatory genes, less is known about the genes suppressed by the stimuli. During microarray analysis of gene expression profiles in lipopolysaccharide (LPS)-stimulated macrophages (TM, unpublished data), we noticed that expression of thioredoxin-interacting protein (Txnip) is dramatically suppressed in response to LPS.

*Txnip* was originally identified as a gene induced by 1,25-dihydroxyvitamin D3, in the leukemia cell line HL-60 [2]. It is a multifunctional gene involved in the cell cycle, cell death, tumorigenesis and metabolism [3]. Its precise molecular function, however, remains elusive. Its gene product interacts with thioredoxin, a regulator of the intracellular redox status, via disulfide bonds at two cysteine residues in the catalytic center, resulting in inhibition of the thioredoxin function [4], [5].

Txnip was also identified as Hyplip1, the disease-causing gene of mouse mutant strain HcB-19, which shares features with human familial combined hyperlipidemia [6]. HcB-19 mice in the fed state exhibit a metabolic profile similar to fasted mice, with increased free fatty acids and ketone bodies in plasma and decreased glucose, suggesting that the Txnip mutation down-regulates the citric acid cycle, sparing fatty acids for triglyceride and ketone body production [6], [7]. Targeted disruption of the Txnip gene in mice also indicated its critical role in energy homeostasis; the mutant mice showed increased fatty acids and decreased glucose in plasma, and enhanced Akt signaling in skeletal muscle and hearts, leading to increased insulin sensitivity and attenulated cardiac hypertrophy [8], [9], [10]. Another report showed that Txnip is also essential for normal development and function of natural killer cells [11]. Txnip expression is induced by high glucose stimulation, mediated by the glucose sensing transcription factor MondoA:Mlx binding to two carbohydrate-response elements (ChoRE) in the promoter [12], [13], [14]. It was reported that Txnip inhibits glucose uptake [9], [15], [16] and more recent report has shown that disruption of Txnip in obese mice significantly ameliorates hyperglycemia, glucose intolerance, and insulin sensitivity [17]. These findings suggest critical roles for Txnip in regulation of cellular glucose metabolism.

Considering implications of inflammation for the metabolic syndromes including obesity, diabetes and arteriosclerosis, and the role of Txnip in the regulation of glucose metabolism, regulation of Txnip expression during inflammatory responses may be involved in homeostasis of inflamed tissues and pathological progression of the diseases. The present study was undertaken to examine regulation of Txnip expression during inflammatory responses and its molecular mechanisms, and showed that Txnip expression driven by MondoA:Mlx was rapidly suppressed upon various inflammatory stimuli.

## Results

### *Txnip* gene expression is down-regulated during inflammatory responses

**Figure 1:**
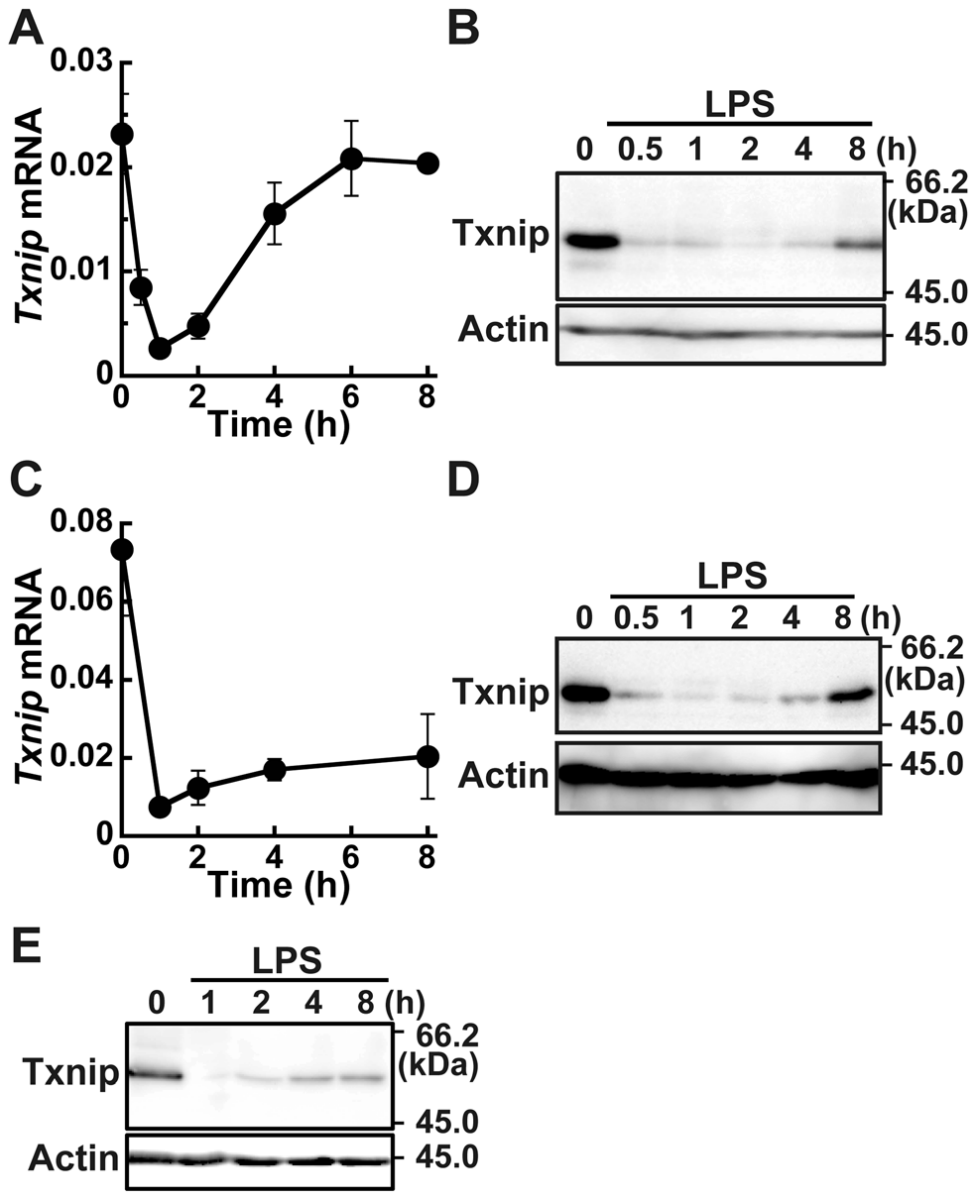
Suppression of *Txnip* expression by LPS. RAW264.7 cells (A and B), BMDM (C and D), or differentiated THP-1 cells (E) were stimulated with 100 ng/ml LPS. Cells were lysed at the indicated time after stimulation. *Txnip* mRNA copy numbers normalized to that of *β-actin* are shown (A and C). Data shown are mean ± S.E. of at least 4 independent experiments. Txnip and β-actin proteins were detected by western blotting (B, D, and E). Data shown are a representative of at least three independent experiments.

In order to examine *Txnip* expression during innate immune responses, the mouse macrophage cell line RAW264.7 cells were stimulated with 100 ng/ml LPS and *Txnip* mRNA expression was examined by quantitative PCR. *Txnip* mRNA was dramatically decreased to approximately 10% of the level in unstimulated cells in 1 h after LPS stimulation. The *Txnip* mRNA level had then returned to the unstimulated level 6 h later (Fig. 1A). The expression level of *Txnip* mRNA did not change in the absence of LPS during the time course (data not shown). Western blotting analysis revealed significantly decreased Txnip protein after LPS stimulation (Fig 1B). Similar analysis of mouse BMDM (Fig. 1C and D) and the human monocyte THP-1 cells differentiated to macrophages (Fig. 1E) demonstrated a rapid decrease of *Txnip* mRNA and protein 1 h after LPS stimulation: the expression levels were then gradually restored following the stimulation.

**Figure 2:**
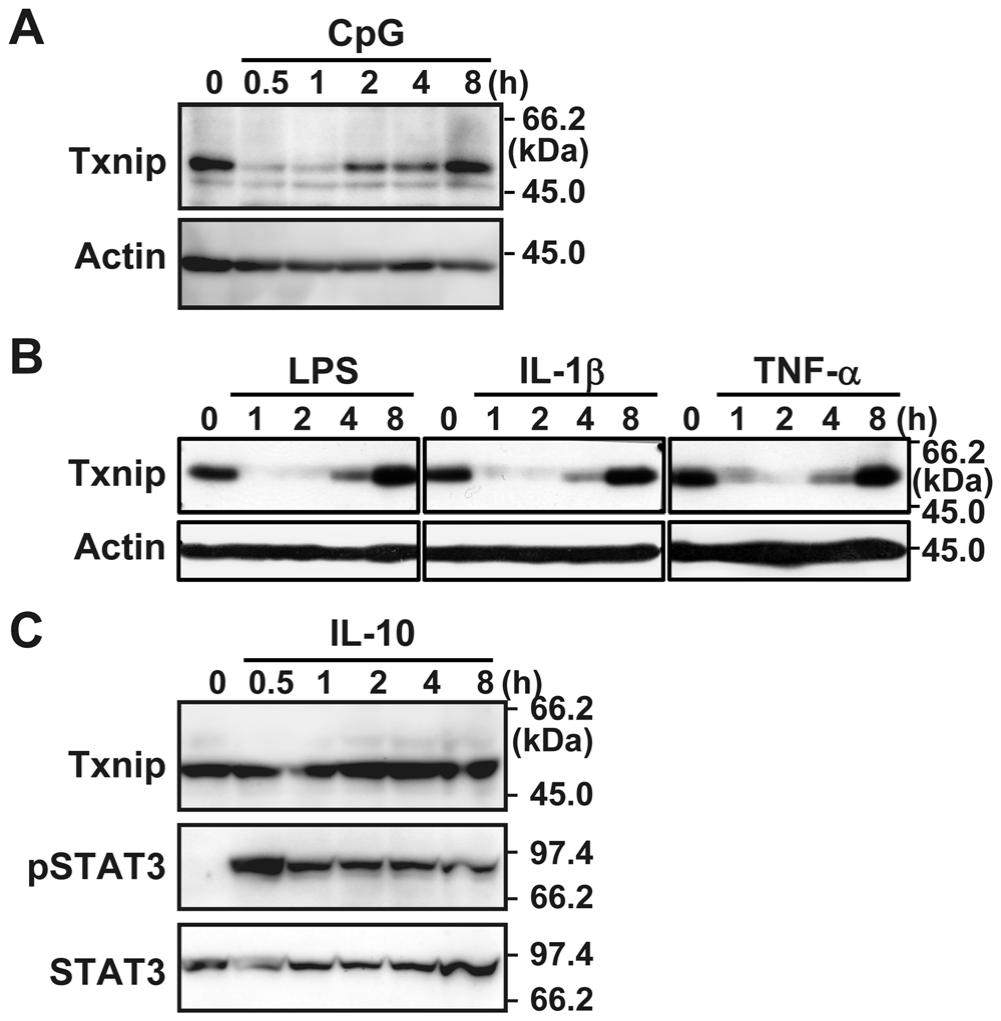
Suppression of *Txnip* expression by proinflammatory stimulation. BMDM (A), NIH3T3 (B), or RAW264.7 (C) cells were stimulated with 2 µM CpG DNA 100 ng/ml LPS, 10 ng/ml IL-1β, 10 ng/ml TNF-α, or 10 ng/ml IL-10. Cells were lysed at the indicated time after stimulation. Txnip, β-actin, phosphorylated STAT3 (pSTAT3), or STAT3 proteins were detected by western blotting. Data shown are a representative of at least three independent experiments.

In addition to the TLR4 ligand LPS, a synthetic triacyl lipopeptide (Pam_3_CSK_4_), poly(I)-poly(C), and CpG DNA, ligands for TLR1/2, 3, and 9, respectively, also induced the decrease of *Txnip* rapidly in BMDM or RAW264.7 cells (Fig. 2A and Fig. S1). Not only these TLR ligands, stimulation with the proinflammatory cytokines IL-1β and TNF-α also resulted in similar suppression of *Txnip* in the fibroblast NIH3T3 cells (Fig. 2B and Fig. S2).

We also examined the effects of the anti-inflammatory cytokine IL-10. Stimulation of RAW264.7 cells with IL-10 elicited phosphorylation of STAT3, but did not affected Txnip expression, further providing evidence for the specific response to proinflammatory stimuli (Fig. 2C).

### Expression and LPS-induced suppression of *Txnip* require ChoRE and CCAAT boxes in the promoter

**Figure 3:**
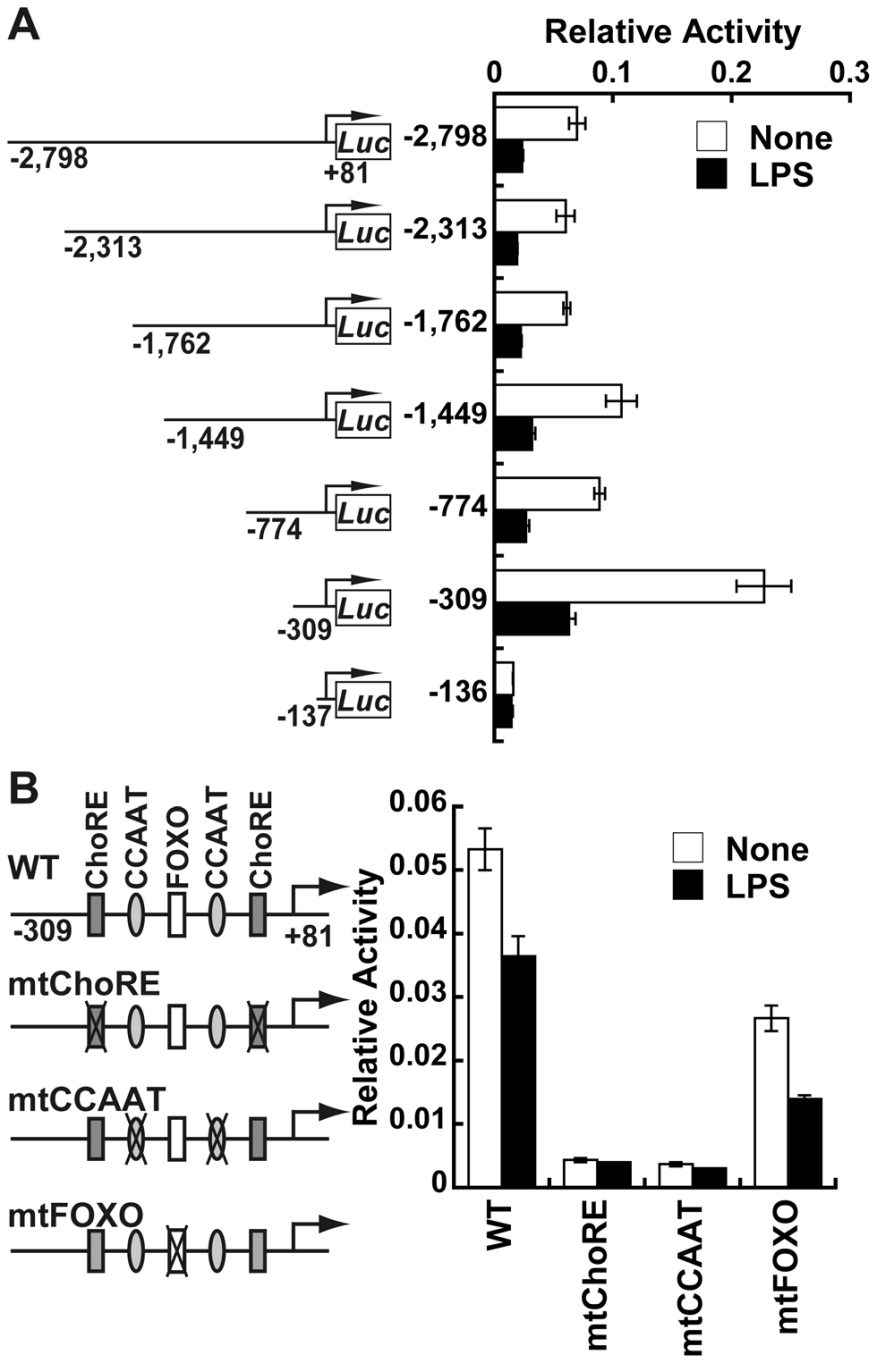
Essential roles for ChoREs and CCAAT boxes in the *Txnip* promoter. (A and B) Luciferase reporter plasmids containing indicated *Txnip* promoter fragments with or without point mutations (mt) at ChoREs, CCAAT boxes, or a FOXO binding site were transfected to RAW264.7 cells together with phRL-TK-luc. Transfected cells were stimulated with or without 100 ng/ml LPS for 4 h, then lysed for luciferase activity measurement. Relative luciferase activities normalized by internal control are shown as mean ± S.E. of triplicate samples. Data shown are a representative of at least three independent experiments.

Next, to identify the pertinent promoter region for the *Txnip* down-regulation after LPS stimulation, the *Txnip* promoter was analyzed. A VISTA plot indicates that a *Txnip* proximal promoter region upto −2.8 kbp from the transcription start site harbors several evolutionarily conserved regions (http://ecrbrowser.dcode.org/xB.php?db=mm9&location=chr3:96361881-96365788) [18]. The promoter activities of various lengths of the *Txnip* promoter region were measured with luciferase reporter plasmids in RAW264.7 cells with or without LPS stimulation (Fig. 3A). Whereas *Txnip* reporters harboring fragments from −309 bp to +81 bp were suppressed after LPS stimulation, a construct with a fragment from −137 to +81 bp was not, and exhibited lower expression even without stimulation. The promoter fragment from −774 to +81 bp and the other longer fragments exhibited lower basal activity, suggesting a repressor binding site in the region between −774 to −309 bp, but those fragments exhibited the similar response to LPS stimulation. These results indicate that a region from −309 to −137 bp is required for basal expression of *Txnip* and that a promoter fragment from −309 bp is sufficient to confer responsiveness to LPS stimulation.

Because the promoter region from −309 bp contains the glucose responding element composed of two ChoREs, two CCAAT boxes, and a forkhead box O (FOXO) binding site, we generated mutant reporters with point mutations at these sites. Constructs with mutations at the ChoREs or CCAAT boxes exhibited severely impaired promoter activity in unstimulated RAW264.7 cells, as in a previous report with other types of cells [13], and hence did not respond to LPS stimulation (Fig. 3B). On the other hand, the mutation at the FOXO binding site did not affect the suppression by LPS. These results demonstrate that the ChoREs and CCAAT boxes of the *Txnip* promoter are essential for expression in unstimulated cells, which is suppressed upon LPS stimulation.

### LPS stimulation induces dissociation of MondoA:Mlx from the *Txnip* promoter

**Figure 4:**
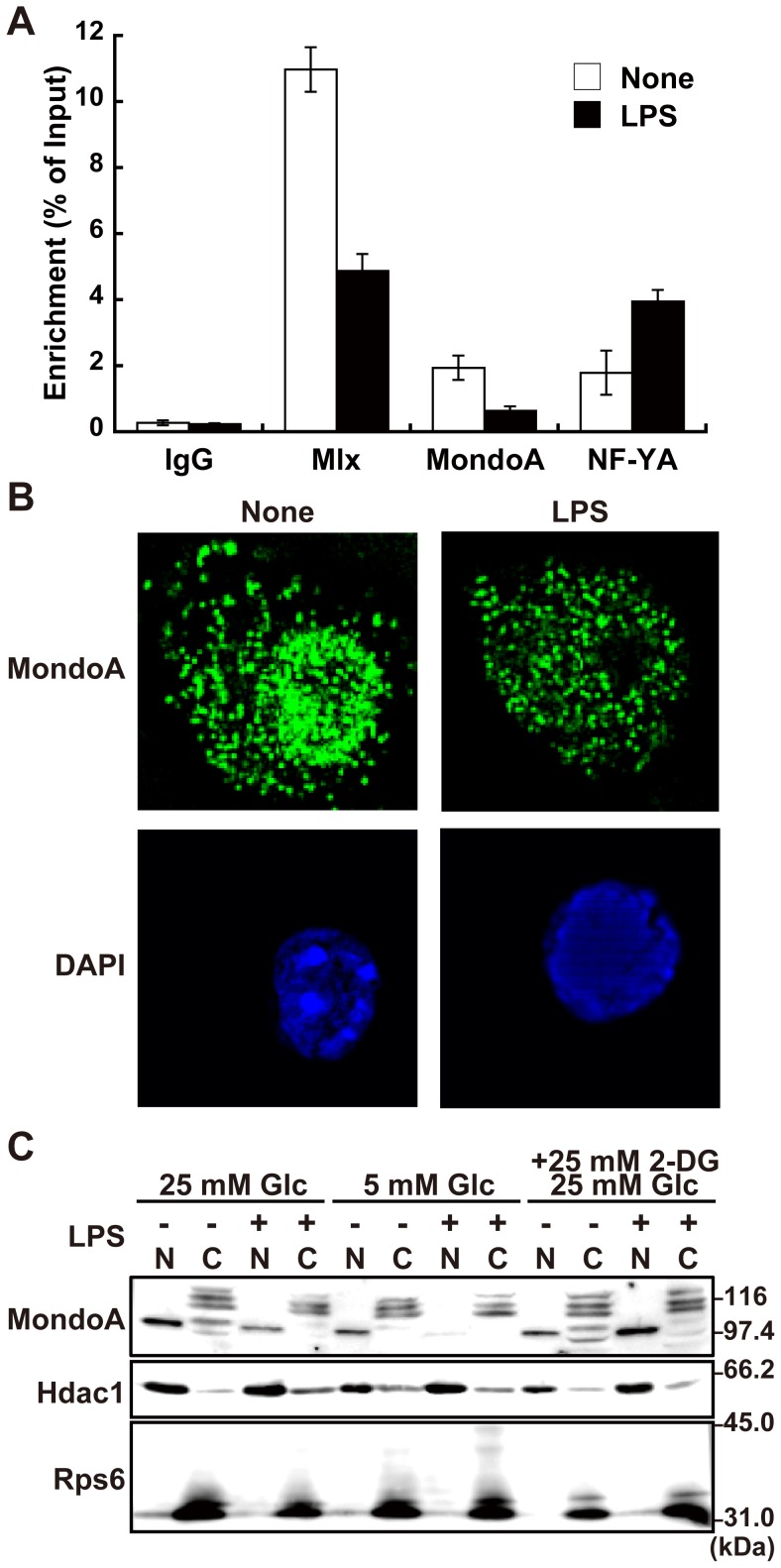
Dissociation of MondoA:Mlx from the *Txnip* promoter on LPS stimulation. (A) Chromatin immunoprecipitation assay was performed to examine occupation of Mlx, MondoA, and NF-YA in *Txnip* promoter region with RAW264.7 cells stimulated with or without 100 ng/ml LPS for 45 min. Enriched DNAs was eluted and *Txnip* promoter region was quantified by quantitative PCR. Data shown are mean ± S.E. of duplicate samples. (B) RAW264.7 cells stimulated with or without 100 ng/ml LPS for 45 min. Cells were fixed, permeabilized, and stained with anti-MondoA antibody and Alexa488-conjugated polyclonal anti-rabbit IgG antibody. Fluorescence microscopic images were obtained and analyzed by three-dimentional deconvolution. (C) Nuclear and cytoplasmic fractions of RAW264.7 cells stimulated with or without 100 ng/ml LPS for 45 min were analyzed by western blotting with anti-*MondoA*, histone deacetylase 1 (*Hdac1*) or ribosomal S6 ribosomal protein (*Rps6*). Data shown are a representative of at least three independent experiments.

It has been reported that for high glucose-mediated induction of the *Txnip*, binding of the glucose sensing transcriptional factor complex MondoA:Mlx and NF-YA to the ChoREs and CCAAT boxes, respectively, is essential [12], [13], [14]. We thus assessed occupation of these transcription factors on the *Txnip* promoter by chromatin immunoprecipitation assays (Fig. 4A). Antibodies against Mlx, MondoA, and NF-YA precipitated significant amounts of the *Txnip* promoter in unstimulated RAW264.7 cells, indicating occupation of these factors on the promoter. LPS stimulation decreased occupancy of the promoter by Mlx and MondoA, whereas NF-YA occupation was increased, indicating specific dissociation of MondoA:Mlx complex from the *Txnip* promoter upon LPS stimulation.

Because induction of *Txnip* expression by high glucose has been reported to be regulated by nuclear accumulation and DNA binding of MondoA [12], [14], subcellular localization of MondoA was investigated. Immunocytochemical analysis with anti-MondoA antibody exhibited punctate staining patterns both in nuclei and cytoplasm in unstimulated RAW264.7 cells. Upon LPS stimulation, the nuclear MondoA decreased without significant alterations of the cytoplasmic staining (Fig. 4B). We further prepared nuclear and cytoplasmic fractions from unstimulated and LPS-stimulated RAW264.7 cells and separately analyzed by western blotting (Fig. 4C). Staining of the marker proteins histone deacetylase 1 (*Hdac1*) and S6 ribosomal protein (*Rps6*) indicated appropriate separation. Anti-MondoA antibody staining showed a single band with mobility consistent with the calculated molecular weight of MondoA (100.8 k) in the nuclear fraction and multiple bands with lower mobility in the cytoplasmic fraction. Consistent with the immunocytochemical analysis, the amount of MondoA in the nuclear fraction was decreased upon LPS stimulation. Similarly, the nuclear MondoA was reduced in cells cultured in lower concentration of glucose (5 mM) compared with cells in normal medium (25 mM glucose). On the other hand, addition of 2-deoxyglucose, a non-metabolizable glucose analog, in medium resulted in inhibition of the loss of the nuclear MondoA in LPS-stimulated cells. The amount of the cytoplasmic bands did not show significant changes and appeared slightly decreased on LPS stimulation.

**Figure 5:**
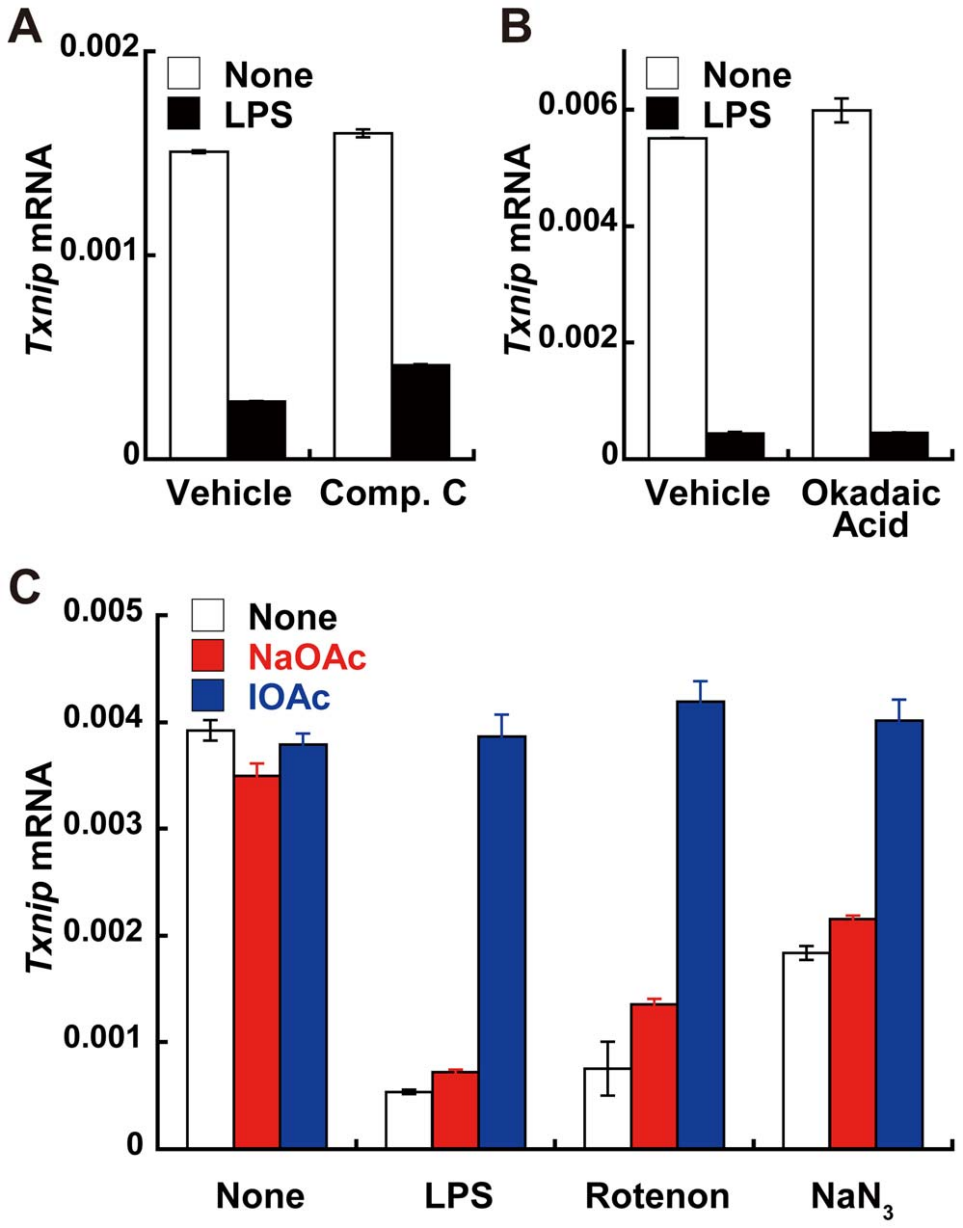
Effects of various inhibitors on *Txnip* suppression in response to LPS. (A and B) RAW264.7 cells were treated with Vehicle (dimethyl sulfoxide in (A) or diethyl ether in (B)), 10 µM compound C (Comp. C), or 50 nM okadaic acid for 1 h, followed by stimulation with 100 ng/ml LPS for 90 min. (C) RAW264.7 cells were treated with 0.5 mM iodoacetate or sodium acetate for 1 h, followed by treatment with 100 ng/ml LPS, 5 mM sodium azide, or 5 µM rotenone for 90 min. *Txnip* mRNA copy numbers normalized to that of β-actin are shown. Data shown are mean ± S.E. of triplicate samples of a representative of at least three independent experiments.

Previous studies have reported that nuclear import/export of ChRE-binding protein, a homologue of MondoA is regulated by its phosphorylation status via AMP kinase and protein phosphatase (PP)2A [19], [20], [21]. However, the AMP kinase and PP2A inhibitors, compound C and okadaic acid, respectively, did not affect *Txnip* suppression by LPS stimulation (Figs. 5A and B). On the other hand, blockage of glyceraldehyde-3-phosphate dehydrogenase activity by iodoacetate, which accumulates glucose metabolites in the glycolytic pathway [22], partially restored *Txnip* mRNA suppression by LPS or the oxidative phosphorylation inhibitors rotenone and sodium azide (Fig. 5C), supporting a role of intracellular glucose metabolites in the regulation of MondoA. Reactive oxygen species scavenger treatment did not affect the *Txnip* suppression by LPS stimulation (data not shown).

## Discussion

The present study demonstrated that *Txnip* gene expression was suppressed by the TLR ligands, such as LPS and CpG DNA, and proinflammatory cytokines IL-1β and TNF-α in mouse and human macrophages and fibroblasts. The observation that the suppression was induced by TNF-α and the TLR3 ligand poly(I)-poly(C), an activator of the TRIF-dependent signaling pathway [23], in addition to TLR ligands and IL-1β that activate the MyD88-dependent signaling [1], suggests that *Txnip* suppression is a universal response to inflammatory stimuli. Because Txnip proteins are constitutively ubiquitinated and turn over rapidly by proteasome-mediated degradation [24], it is likely that *Txnip* expression is down-regulated at least at the transcriptional level in response to LPS. A previous report showed that serum stimulation of fibroblasts resulted in inhibition of both transcription and translation of *Txnip* [25]. Considering rapid and dramatic down-regulation of both mRNA and protein expression levels, LPS-induced *Txnip* suppression may involve active degradation of *Txnip* mRNA and proteins on the stimulation. In fact, stimulation of Txnip degradation by cAMP was reported previously [26].

It was revealed that ChoREs and CCAAT boxes in the *Txnip* promoter region are critical for basal expression of *Txnip* in macrophages. Both MondoA and Mlx, which bind to ChoREs, were associated with the *Txnip* promoter in unstimulated cells, but dissociated upon stimulation with LPS. It was reported that *Txnip* expression was dramatically suppressed by inhibitors of mitochondrial oxidative phosphorylation such as rotenone, antimycin and sodium azide in HeLa cells [22]. The suppression induced by these inhibitors is mediated by the dissociation of MondoA:Mlx from the *Txnip* promoter, probably caused by a decrease of glucose metabolites due to glycolysis activation to replenish intracellular ATP [22]. *Txnip* suppression by these inhibitors is similar to that by LPS stimulation in both time course and degree. The finding that an inhibitor of glyceraldehyde-3-phosphate dehydrogenase, iodoacetate, which induces the accumulation of glucose metabolites, partially restored the *Txnip* suppression by LPS is consistent with a hypothesis that MondoA:Mlx senses a decrease in glucose metabolite concentration associated with LPS stimulation. It has been reported that *Txnip* expression is also suppressed in response to insulin [15] and glutamine [27], both of which stimulate the glycolytic rate.

MondoA has been reported to shuttle between nucleus and cytoplasm and glucose stimulates its nuclear accumulation [12], [14], [28], [29]. In addition to the nuclear fraction, we detected multiple bands with slightly slower mobility than nuclear MondoA in the cytoplasmic fraction. Precise identities of these protein bands remain unclear although these may represent splicing variants and/or phosphorylated forms of MondoA. Upon LPS stimulation, a decrease of nuclear MondoA was observed both in immunocytochemical analyses and immunoblotting. We, however, failed to detect any increased cytoplasmic band that accounts for its nuclear export upon stimulation. Thus, the possibility that a mechanism other than glucose-induced translocation regulates MondoA in response to LPS is not excluded.

Nevertheless, it is tempting to hypothesize metabolic changes during inflammatory responses in relation to its physiological consequences. It is well known that T-cell activation via T-cell receptor shifts energy metabolism from oxidative phosphorylation to glycolysis, resulting in T-cell proliferation and cytokine production [30]. Recent reports have indicated that inhibition of glycolysis with low-dose 2-deoxyglucose polarized to Treg differentiation in Th17 differentiation conditions [31], [32]. This suggests that the cells switch energy metabolism in response to various stimulations for normal functions. It has been reported that classical and innate activation of macrophages increases glycolytic flux [33] and that AMP kinase is activated in response to LPS [34].

Although the precise molecular mechanisms of LPS-induced glycolytic activation remain elusive, it appears that inhibition of glycolysis might affect expression of proinflammatory genes. For example, treatment with 2-deoxyglucose, a hexokinase inhibitor, severely inhibits IL-1β and IL-6 transcription [35]. Thus, our results suggest that *Txnip* suppression by LPS stimulation might reflect the physiological regulation of energy metabolism that accompanies the innate immune response. In view of physiological and pathological relations between inflammation and various disorders including metabolic syndromes, investigations on the metabolic changes during inflammatory processes would give more insights into understanding of bidirectional regulations of inflammation and metabolisms.

## Materials and Methods

### Ethics Statement

Animal experiments were performed in compliance with the animal care and use guidelines of the Institutional Animal Care and Use Committee of Tohoku University. The animal protocol was approved by the same committee.

### Cells and reagents

RAW264.7 (ATCC^®^ Number, TIB-71™) and NIH3T3 were cultured in Dulbecco's modified Eagle's medium containing 25 mM glucose, 10% heat inactivated fetal calf serum, and 100 U/ml penicillin and 100 µg/ml streptomycin. Bone marrow-derived macrophages (BMDM) were prepared from C57BL/6 mice and cultured for 1 week in the same medium containing 10% L929 culture supernatant. THP-1 cells were cultured in RPMI-1640 containing 10% fetal calf serum and were differentiated by stimulation with 100 nM phorbol 12-myristate 13-acetate for 3 h, followed by 3 days in culture.

LPS from *Escherichia coli* O111:B4 and phosphorothioate-linked CpG DNA (TCCATGACGTTCCTGACGTT) were obtained from Sigma-Aldrich Japan K. K. (Tokyo, Japan). Mouse interleukin (IL)-1β and tumor necrosis factor (TNF)-α were from Jena Bioscience GmbH (Jena, Germany). IL-10 was from R&D Systems (Minneapolis, MN). Anti-*Txnip* (JY-2) and anti-MLX (AF4186) antibodies were purchased from Medical Biological Laboratory Co. Ltd. (Nagoya, Japan) and R&D Systems, respectively. Anti-STAT3 (79D7), phosphorylated STAT3 (Y705) (3E2), and anti-S6 ribosomal protein (5G10) antibodies were from Cell Signaling Technology, Inc. (Danvers, MA). Anti-MondoA (sc-133397), anti-histone deacetylase 1 (sc-7872), and anti-nuclear factor (NF)-YA (sc-100779) antibodies were from Santa Cruz Biotechnology, Inc. (Santa Cruz, CA).

### RNA extraction and quantitative PCR

Total RNA was extracted using RNAIso (Takara Bio Inc., Otsu, Japan), and cDNA was generated using the High Capacity cDNA Reverse Transcription kit (Applied Biosystems, Foster City, CA). Quantitative real-time PCR assays were performed using a LightCycler (Roche Diagnostics Corporation, Indianapolis, IN) with Syber Premix Ex Taq (Takara Bio) and the following oligonucleotide primers: *Txnip*, AGTCGAATACTCCTTGCT and CTCAGGGGCGTACATA; *β-actin*, GATGACCCAGATCATGTTTGA and GGAGAGCATAGCCCTCGTAG.

### Luciferase assay

DNA fragments of the *Txnip* promoter region was cloned into the *SacI and XhoI* restriction sites located upstream of the *luc2cp* gene in the pGL4.12 plasmid (Promega Corporation, Madison, WI). Point mutations in ChoRE, CAATT [13] and FOXO1 elements (GTAAACAAG to GcAgACgAG) were introduced by PCR-based site-directed mutagenesis. Using Lipofectamine™ LTX (Invitrogen), reporter construct was cotransfected with phRL-TK (Promega) into RAW264.7 cells; cells were stimulated 24 h later with LPS for 4 h, and then lysed. Luciferase activity was measured using the Dual-Luciferase Reporter Assay system (Promega).

### Western blotting analyses

Nuclear and cytoplasmic fractions were obtained with a NE-PER Nuclear and Cytoplasmic Extraction Reagent Kit (Thermo Fisher Scientific Inc., Rockford, IL). The fractions were subjected to western blotting analyses with indicated antibodies. Reacting bands were visualized by chemiluminescence by incubation with Immobilon Western Chemiluminescent HRP Substrate (Millipore).

### Chromatin immunoprecipitation assay

Chromatin immunoprecipitation was carried out using indicated antibodies as previously described [36]. Following immunoprecipitation, enriched DNA was eluted and quantified by quantitative PCR using primers for the *Txnip* promoter, CCCAAGAGGAGTCCCCTGGATG and GTCAAGCGGCTGCCGGAAACGG.

### Immunocytochemistry

Cells were fixed with 1% paraformaldehyde and permeabilized with 0.2% Triton-X for 10 min. Cells were blocked with fetal calf serum and incubated with anti-MondoA antibody overnight at 4°C and then with anti-rabbit polyclonal IgG conjugated with Alexa488 (Invitrogen). Cells were stained with 4′,6-diamidino-2-phenylindole and observed under fluorescence microscopy with the Leica AF6000 fluorescence imaging system (Leica Microsystems Inc., Wetzlar, Germany) and analyzed with the MetaMorph software (Molecular Devices, LLC, Sunnyvale, CA).

## Supporting Information

Figure S1 **Suppression of** ***Txnip*** **expression by TLR ligands.** RAW264.7 cells were stimulated with 1 µM CpG DNA, 100 ng/ml Pam_3_CSK_4_, or 10 µg/ml poly(I)-poly(C) (poly I:C) in the presence of 100 U/ml polymyxin B. Cells were lysed at the indicated time after stimulation. *Txnip* mRNA copy numbers normalized to that of β-actin are shown. Data shown are a representative of at least three independent experiments.
(TIF)

Figure S2 **Suppression of** ***Txnip*** **expression by proinflammatory cytokines.** NIH3T3 cells were stimulated with 100 ng/ml LPS, 10 ng/ml TNF-α, or 10 ng/ml IL-1β. Cells were lysed at the indicated time after stimulation. *Txnip* mRNA copy numbers normalized to that of β-actin are shown. Data shown are a representative of at least three independent experiments.
(TIF)
