# Supplementary figures and images for: Thioredoxin-Interacting Protein Gene Expression via MondoA Is Rapidly and Transiently Suppressed during Inflammatory Responses
Source: PLoS One. 2013 Mar 8;8(3):e59026. doi: 10.1371/journal.pone.0059026 (PMC3592832; doi:10.1371/journal.pone.0059026)

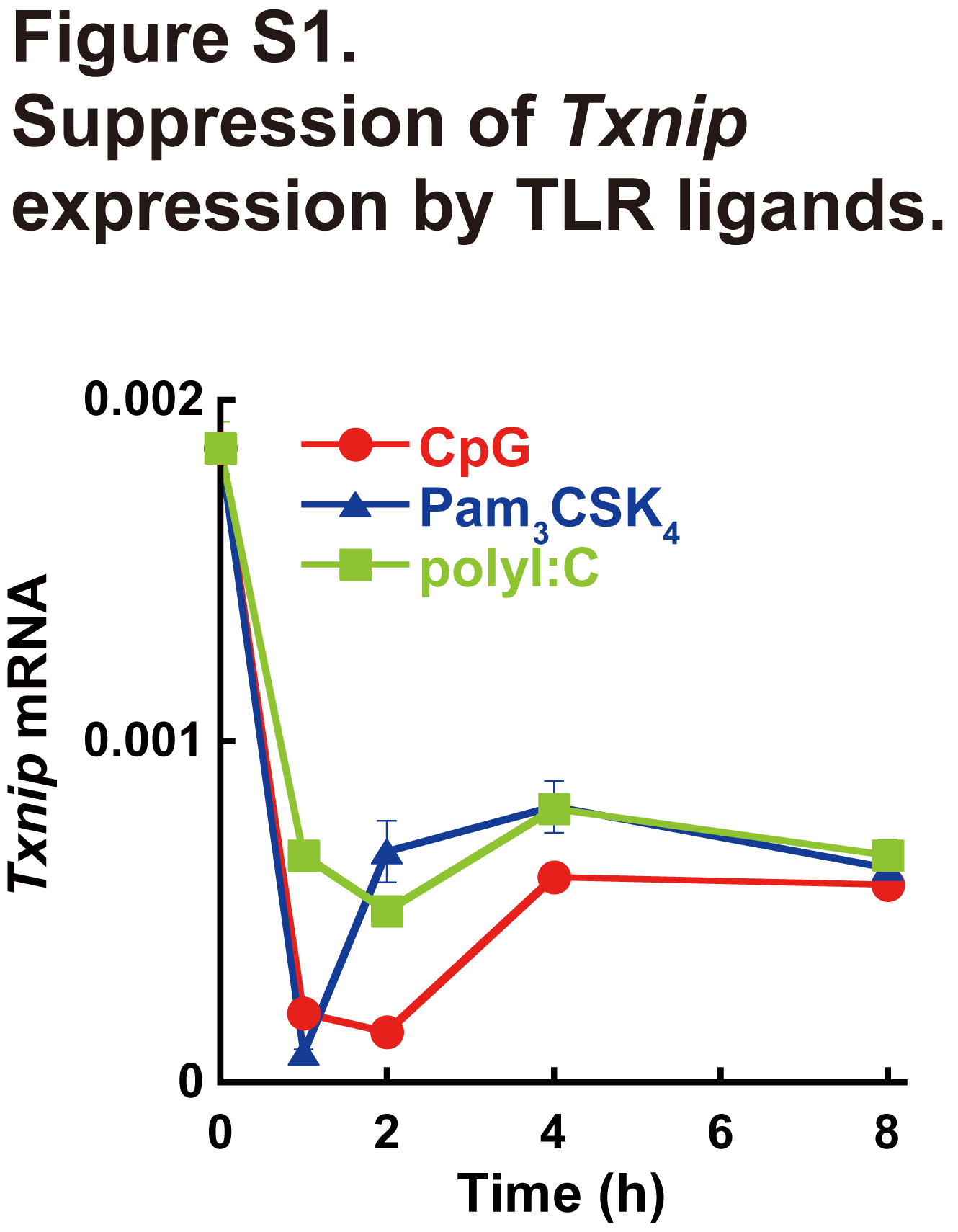

Supplement: Figure S1 — Suppression of Txnip expression by TLR ligands. RAW264.7 cells were stimulated with 1 µM CpG DNA, 100 ng/ml Pam3CSK4, or 10 µg/ml poly(I)-poly(C) (poly I:C) in the presence of 100 U/ml polymyxin B. Cells were lysed at the indicated time after stimulation. Txnip mRNA copy numbers normalized to that of β-actin are shown. Data shown are a representative of at least three independent experiments. (TIF) [file pone.0059026.s001.tif]

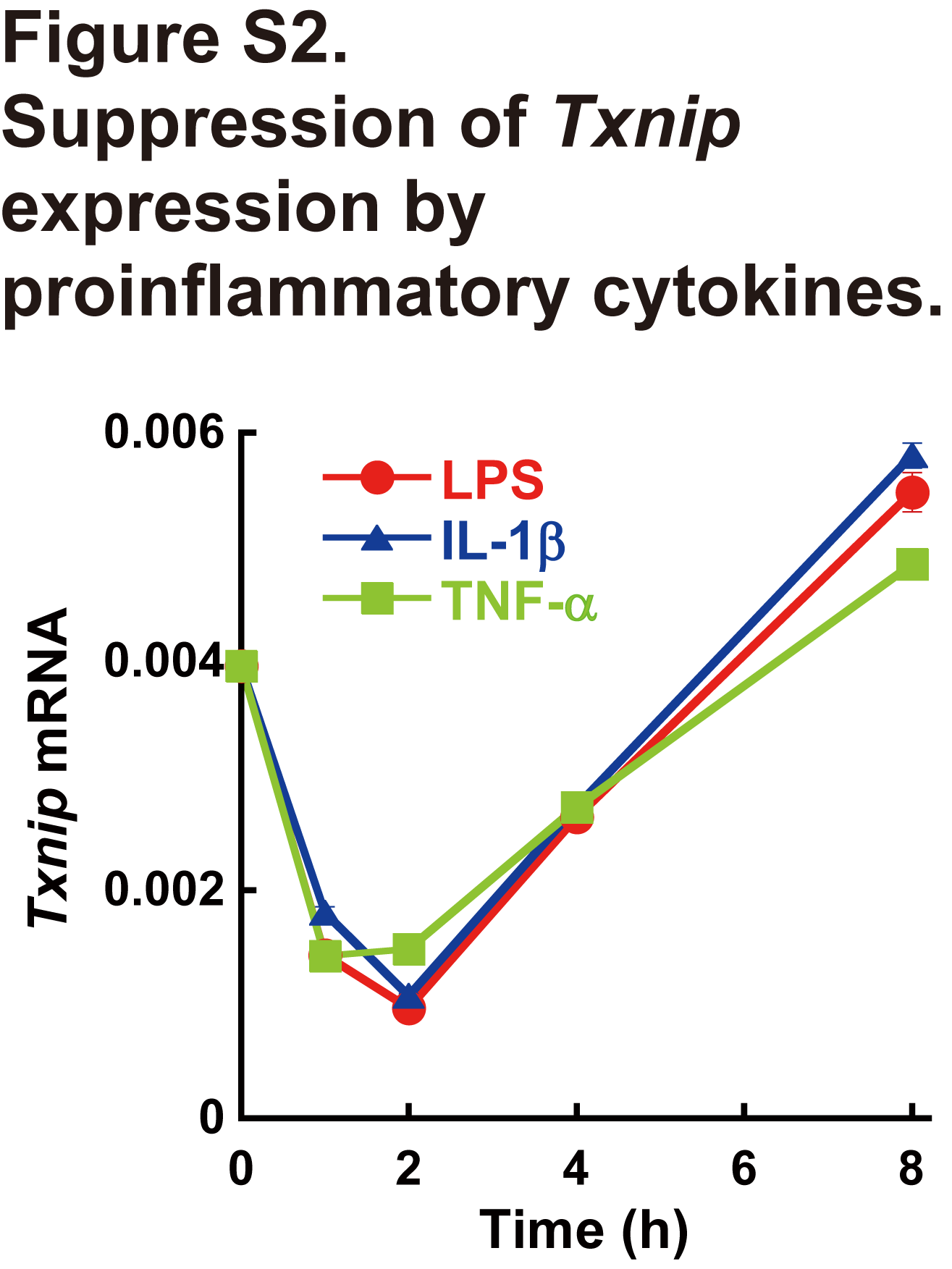

Supplement: Figure S2 — Suppression of Txnip expression by proinflammatory cytokines. NIH3T3 cells were stimulated with 100 ng/ml LPS, 10 ng/ml TNF-α, or 10 ng/ml IL-1β. Cells were lysed at the indicated time after stimulation. Txnip mRNA copy numbers normalized to that of β-actin are shown. Data shown are a representative of at least three independent experiments. (TIF) [file pone.0059026.s002.tif]
